# Missing depth cues in virtual reality limit performance and quality of three dimensional reaching movements

**DOI:** 10.1371/journal.pone.0189275

**Published:** 2018-01-02

**Authors:** Nicolas Gerig, Johnathan Mayo, Kilian Baur, Frieder Wittmann, Robert Riener, Peter Wolf

**Affiliations:** 1 Sensory-Motor Systems Lab, Department of Health Science and Technology, Swiss Federal Institute of Technology, Zurich, Switzerland; 2 Spinal Cord Injury Center, University Hospital Balgrist, University of Zurich, Zurich, Switzerland; 3 Rehabilitation Engineering Lab, Department of Health Science and Technology, Swiss Federal Institute of Technology, Zurich, Switzerland; University of Illinois at Urbana-Champaign, UNITED STATES

## Abstract

**Background:**

Goal-directed reaching for real-world objects by humans is enabled through visual depth cues. In virtual environments, the number and quality of available visual depth cues is limited, which may affect reaching performance and quality of reaching movements.

**Methods:**

We assessed three-dimensional reaching movements in five experimental groups each with ten healthy volunteers. Three groups used a two-dimensional computer screen and two groups used a head-mounted display. The first screen group received the typically recreated visual depth cues, such as aerial and linear perspective, occlusion, shadows, and texture gradients. The second screen group received an abstract minimal rendering lacking those. The third screen group received the cues of the first screen group and absolute depth cues enabled by retinal image size of a known object, which realized with visual renderings of the handheld device and a ghost handheld at the target location. The two head-mounted display groups received the same virtually recreated visual depth cues as the second or the third screen group respectively. Additionally, they could rely on stereopsis and motion parallax due to head-movements.

**Results and conclusion:**

All groups using the screen performed significantly worse than both groups using the head-mounted display in terms of completion time normalized by the straight-line distance to the target. Both groups using the head-mounted display achieved the optimal minimum in number of speed peaks and in hand path ratio, indicating that our subjects performed natural movements when using a head-mounted display. Virtually recreated visual depth cues had a minor impact on reaching performance. Only the screen group with rendered handhelds could outperform the other screen groups. Thus, if reaching performance in virtual environments is in the main scope of a study, we suggest applying a head-mounted display. Otherwise, when two-dimensional screens are used, achievable performance is likely limited by the reduced depth perception and not just by subjects’ motor skills.

## Introduction

Goal-directed reaching towards an object is an essential movement to enable grasping, moving, or manipulating objects [1]. The ability to perform goal directed reaching is often impaired due to reduced muscle function or muscular control following spinal cord injury or stroke [2].

In the last two decades, robots and instrumented setups have been developed to enhance upper-limb rehabilitation therapy [3]. Robots or instrumented setups equipped with sensors can be used to augment therapy exercises with virtual reality. Virtual reality can provide augmented performance feedback to increase learning [4] and can increase motivation [5, 6]. Additionally, reproducible conditions for quantitative assessments of movements can be realized using virtual reality and sensors [2, 7]. Such quantitative assessments could lead to more objective grading of impairment or function, which would result in a better understanding of the rehabilitation process and eventually in an improved planning of individual therapy.

However, virtual reality may limit natural perception of goals or movement targets, which could affect both movement planning and execution. These drawbacks may cause the trained movements to differ from natural movements resulting in movement artifacts or artificial movement patterns. Such artificial movement patterns are undesirable, because they could reduce translation from learning during rehabilitation into daily living and reduce informativeness of quantitative assessments.

While drawbacks of robots and sensors are specific to the hardware being employed, the virtual reality and display principles are the same for a wide range of different rehabilitation robots. Typically, a three-dimensional virtual world with objects that should be grasped or collected by the user are displayed on a two-dimensional computer screen or projector, e.g. in [8, 9]. Therefore, we wanted to quantify the limitations of such typical virtual reality setups on natural reaching movements.

Natural reaching movements in three-dimensional space require planning and perception of the target’s location. While visual perception of a target’s location in the coronal plane is relatively simple due to arrangement of photo-receptors on the retina, humans need to rely on different visual depth cues to perceive the distance to a target [10]. Using virtual reality, provision of these natural depth cues is limited. Some depth cues can be artificially recreated with respective renderings in the virtual world, e.g. aerial perspective, linear perspective, motion parallax, occlusion, shadows, texture gradients, or retinal image size with objects of known size. Other depth cues are limited by two-dimensional computer screens, e.g. stereopsis requires an individual image per eye. Oculomotor depth cues might not be realizable at all, since the eye’s lens focus is dependent on the distance to the display and not to the target.

Research on egocentric distance perception in virtual reality covers the perception of distance between a virtual object and the user (see [11] for a review). However, research in this area has been performed in a fashion mostly isolated from an action-perception cycle. It remains unclear, whether egocentric distance perception is required for a closed action-perception cycle, e.g. when training and assessing reaching function with virtual reality: Potentially, depth relations between objects in virtual reality provide sufficient information for the human brain to create a visuomotor mapping to perform reaching movements with natural movement kinematics. The finding that the relevance and relative importance of depth cues depends on the task at hand seems to be supported by both research in egocentric distance perception [11] and experiments with closed action-perception cycle (e.g. [12]). For training or assessing three-dimensional reaching movements, guidelines for designing virtual environments are missing.

While rehabilitation setups typically use two-dimensional screen setups, a new generation of head-mounted display (HMD) systems have reached the consumer market for entertainment. Modern head-mounted display systems (e.g. HTC Vive *[High Tech Computer Corporation, Taoyuan, Taiwan]*, Oculus Rift *[Oculus VR LLC, Menlo Park, California, USA]*, Sony PlayStation VR *[Sony Corporation, Minato, Tokyo, Japan]*, provide affordable solutions for stereopsis, a large field of view, and head tracking for viewing angle and position dependent rendering of the virtual world. Whether these systems provide the potential to enhance rehabilitation therapy by reducing potential limitations of typical display setups has not been investigated.

To assess reaching function or impairments quantitatively, raw sensor data or hand movement trajectories have to be evaluated, e.g. smoothness metrics are used to describe the efficiency of a movement or the quality of motor control, often applied with the goal of measuring recovery [7]. Such metrics were based on theoretical assumptions, e.g. that natural, ideally energy-efficient point-to-point reaching movements would be straight and would consist of a single speed peak with only one acceleration and one deceleration phase. While these metrics would provide a simple interpretation due to their theoretical limit, knowing how movements were affected and limited in these metrics is critical for their interpretation, e.g. limitations arising from the setup must be known to judge impairment or function. If healthy persons did not perform straight reaching movements with a single speed peak in a given setup, an extensive normative data set would be required to judge patients’ reaching movements.

This work aimed to quantify effects on reaching performance and quality of movement metrics in healthy subjects caused by limitations in depth cue rendering. We wanted to show the potential between current state-of-the art rehabilitation setups and what is feasible with current consumer hardware. Additionally, we wanted to elaborate practical suggestions for the design of virtual reality for three dimensional reaching movement therapy. We advanced these goals by comparing reaching performance and quality of movement of healthy subjects between different screen and head-mounted display conditions.

## Materials and methods

### Experimental setup

**Fig 1:**
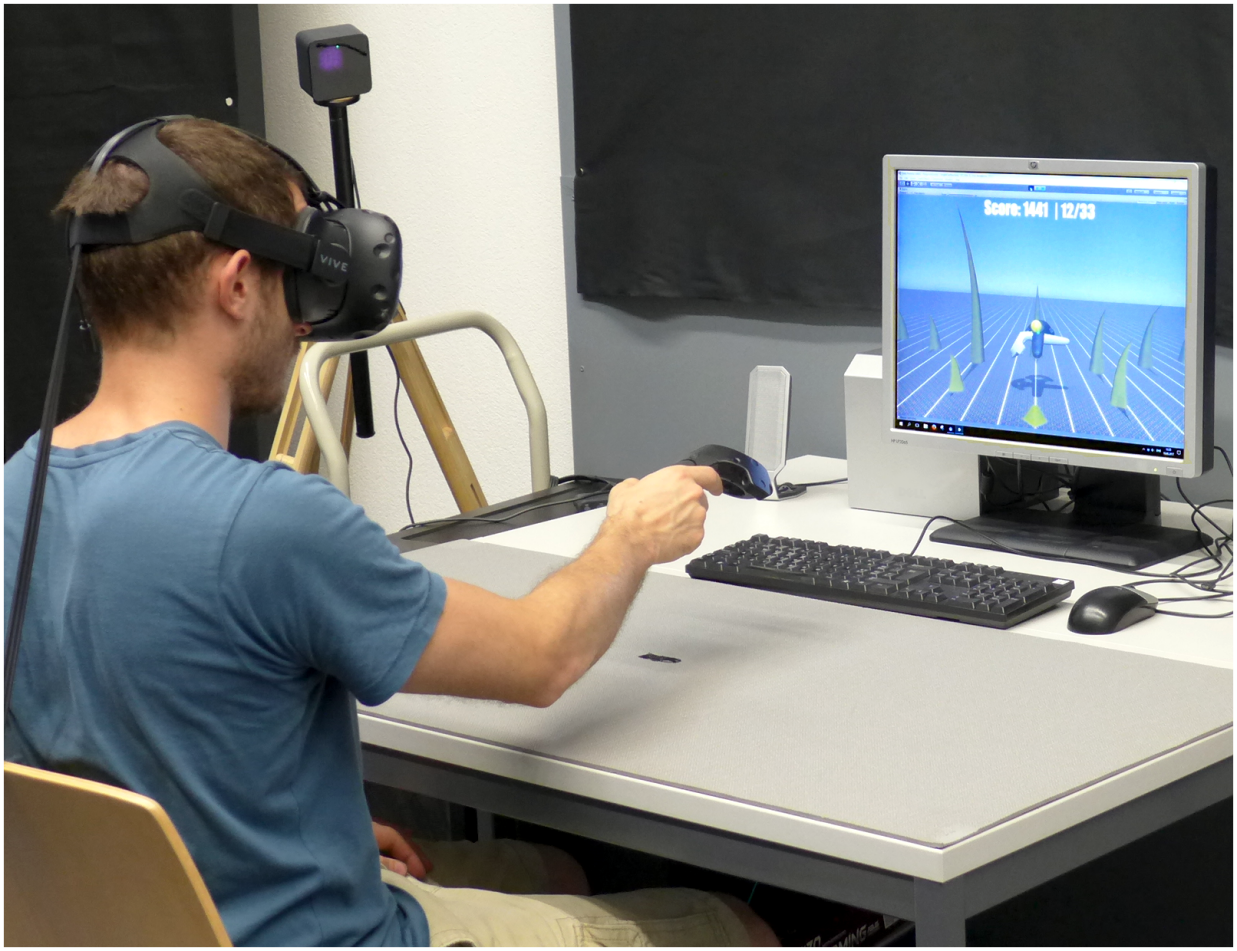
Subject using the experimental setup. The subject sat at the cushioned table with resting position indicated with a stripe in the table center. Handheld (right hand) and head-mounted display (head) were tracked by the Lighthouse with one base station visible in the back. The desktop monitor was located and oriented as used during experiments without the head-mounted display. A clearer picture of the virtual scene with better contrast and colors is provided in Fig 2. The depicted individual is the corresponding first author. The individual in this manuscript has given written informed consent (as outlined in PLOS consent form) to publish these case details.

Subjects were seated at a controlled position, centered in front of a cushioned table (Fig 1). The experiments were realized with an HTC Vive *[High Tech Computer Corporation, Taoyuan, Taiwan]* virtual reality system driven by a desktop computer (Microsoft Windows 10 *[Microsoft Corporation, Redmond, Washington State, USA]*, Intel i5-6600K CPU @3.5 GHz *[Intel Corporation, Santa Clara, California, USA]*, Nvidia GTX 1080 GPU *[Nvidia Corporation, Santa Clara, California, USA]*). The HTC Vive consists of a head-mounted display (1080x1200 pixels per eye @90 Hz, 110° horizontal field of view, 0.6 kg), a handheld controller (0.2 kg), and the Valve Lighthouse *[Valve Corporation, Bellevue, Washington State, USA]* optical tracking system (Fig 1). According to the manufacturer, Lighthouse offers sub-millimeter accuracy and between 250 Hz to 1 kHz update rates [13]. Subjects that did not use the head-mounted display still operated the same HTC Vive handheld, but the virtual environment was displayed using a desktop monitor (HP LP2065 *[HP Incorporated, Palo Alto, California, USA]*, 20”, 1600x1200 @85 Hz). The desktop monitor was located at a distance of 0.9 m from the user-facing table edge. This screen distance was chosen to be similar to three dimensional robotic rehabilitation setups [8], to avoid subjects hitting the screen while their arm was fully extended. Virtual reality and raw data logging (at approximately 1 kHz) were realized in a custom-made Unity *[Unity Technologies, San Francisco, California, USA]* game using the OpenVR library [14].

### Task and protocol

The subjects were required to move a virtual, pink cylindrical pointer (0.05 m length, 0.01 m diameter) from a resting position on the table towards a target sphere and keep a part of the pointer inside the individual target sphere for two seconds. The target sphere (0.04 m diameter) was yellow and semitransparent. If any part of the pointer was within the target sphere, the cylindrical pointer was displayed in green instead of pink. The cylindrical pointer was located on the track-pad of the handheld and was pointing in a skewed upward-forward direction. Location and orientation were chosen to approximate the tip of the thumb for a nominal hand size and grip of the handheld.

Subjects were instructed to perform the task as quickly as possible. The controlled spatial tolerance given by the 0.04 m sphere was considered to control speed-accuracy trade-off and justify time to completion as a reasonable primary outcome metric. In total, 33 targets were presented to each subject. Targets were distributed throughout a quarter-sphere of 0.32 m radius. The first four targets were placed at the extremes of the work-space and served to test the subjects’ range of motion and familiarize them with the task procedure. All subjects received the same targets in the same order.

At the start of each trial, the target sphere was displayed in starting position. Once the subject reached the starting position, a 15 s countdown was started. The first 10 s of this countdown were provided as a resting period before presenting the next target to avoid fatigue. During this first 10 s, the countdown from 15 to 6 was visually displayed. The next target was presented 5 s before the countdown ran out, to allow the subject to locate the target in space and plan the movement. To avoid occlusion of the scenery, the starting countdown from 5 to 0 was not displayed visually. Instead the last 3 s (3, 2, 1) were indicated with 440 Hz sounds of 0.2 s duration each, and the start sound at the end of the countdown with a frequency of 880 Hz and of 0.2 s duration, mimicking the start sounds commonly used for virtual car races. The start sound was accompanied by a small vibration impulse realized with the handheld. In case of a false start, i.e. moving out of a 0.04 m diameter sphere around the starting position before the start signal, the trial was excluded from analysis. After false start or task completion, the start position was indicated with the target sphere again. Once the subject returned to the start position, the next 15 s countdown was started.

To keep subjects motivated, they received performance feedback for their last reaching movement during resting time. The score *S*_*i*_ of movement *i* was calculated as *S*_*i*_ = 300 · *d*_*i*_/Δ*t*_*i*_, where *d*_*i*_ was the straight-line distance between start and target position in meters, and Δ*t*_*i*_ the time to target in seconds (i.e. time to task completion without the last two seconds of dwell time). The gain was chosen such that a two to three-digit integer score would typically be achieved.

### Kinematic evaluation

#### Main outcome: Time to target

The experiment was designed such that the time required to reach the target was the primary outcome directly reflecting the inverse of reaching performance. Start time-point, stop time-point (when the successful 2 s dwell time started), and time to target (the difference between those) were logged directly from the events in the custom-made Unity game.

#### Secondary outcomes: Number of speed peaks and hand path ratio

Number of speed peaks and hand path ratio are measures that were applied in studies to assess movement smoothness, amount of sub-movements, or quality of motor control [7]. These secondary outcomes were evaluated in custom written Matlab scripts from the raw position data that was logged in a separate thread of the custom made Unity game. For evaluation, the raw data was segmented into individual movements using the start time point and stop time point logged from the game events.

In this work, the number of peak speeds was evaluated as the number of peaks in a smoothed speed signal that had a prominence (i.e. peak height of local maximum compared to adjacent local minima) of more than 5% of the total span observed in the smoothed speed signal of this movement. The smoothed speed signal was obtained by applying the same low-pass filter (finite impulse response, equiripple design, passband frequency 0.12 Hz, stopband frequency 31 Hz, passband ripple 1 dB, stopband attenuation 60 dB) forwards and backwards on the Euler-norm of velocity. Velocity was calculated as the sample-wise difference of raw positions divided by sample time. The automated peak speed detection might fail to identify an eventual last speed peak in case the deceleration was performed during the dwell time inside the target sphere.

Hand path ratio was obtained by dividing the subject’s performed movement length over the reference target distance. Performed movement length was obtained by summarizing the Euler-norm of sample-wise difference of raw positions. A hand path ratio of exactly 1 could reflect a perfect straight line from center of the start position sphere to center of the target sphere. Since shorter movements from the inside of the start sphere to the inside of the target sphere than from center to center are possible, hand path ratios smaller than 1 are also possible.

### Virtual environment and experimental groups

The virtual environment for the reaching movement was designed to provide a minimal amount of rendered objects and distractions, while providing all necessary objects for realizable visual depth cues (see e.g. [10] for an overview on depth cues). In contrast with virtual environments used in rehabilitation training, which are often artistic and enriched with motivating elements, this minimalist approach for the virtual environment was chosen to maximize clarity of the applied depth cues, and to provide a neutral assessment environment. A neutral assessment environment was assumed to be beneficial in reducing motivation differences between subjects reacting different to contextual elements. Additionally, neutral objects with realistic scaled sizes were not used in the background scenery since virtual environments used in rehabilitation often lack such objects, e.g. in neutral assessment environments or in purely motivational training games. Five experimental groups were investigated in a parallel block design, which received different visual depth cues (group characteristics provided in Table 1).

**Table 1:** Realized depth cues and experimental groups.

Depth cue	Implementation	*Screen State*	*Screen Minimal*	*Screen Full*	*HMD Minimal*	*HMD Full*
**Aerial perspective**	Virtual fog.	✔		✔		✔
**Linear perspective**	White parallel lines on the scenery ground.	✔		✔		✔
**Motion parallax**(scenery motion)	Constant virtual wind moving blades of grass, closer blades of grass appear to be moving more.	✔		✔		✔
**Occlusion**	Blades of grass, that may partially occlude each other, target, or pointer.	✔		✔		✔
**Shadows**	Shadows of grasses, pointer, target, handhelds, and ghost handheld.	✔		✔		✔
**Texture gradients**	Grey texture at scenery ground, where a more detailed rendering is visible closer to the subject.	✔		✔		✔
**Retinal image size** (absolute)	Objects of absolutely known size at both, pointer position by showing virtual black handheld, and at target position by white ghost handheld (Fig 2, right).			✔		✔
**Retinal image size** (relative)	Unrelated objects of relatively known size, constant sized pointer and constant sized target sphere.	✔	✔	✔	✔	✔
**Motion parallax** (subject motion)	Virtual scene is rendered from the current viewing position and angle, which can be tracked by the HMD, close objects seem to move more.				✔	✔
**Stereopsis**	Individual screens for each eye in the HMD providing different images for both eyes.				✔	✔

**Fig 2:**
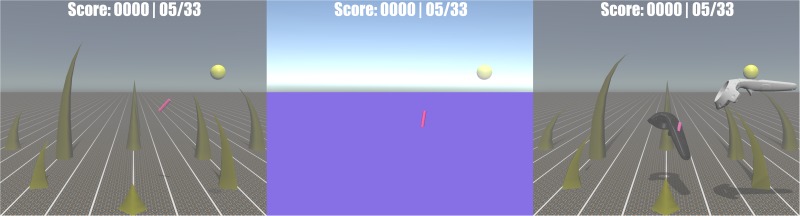
Comparison of the different virtual reality renderings. All three conditions show the pink pointer moving towards the yellow target sphere of the 5^th^ target. The state-of-the art rendering (left) of subjects in the *Screen State* includes fog, linear perspective, grasses, shadows, and textures. The minimal rendering (middle) of subjects in the groups *Screen Minimal* and *HMD Minimal* lacks these depth cues. The full rendering of subjects in the groups *Screen Full* and *HMD Full* additionally contains the rendered handheld and a ghost handheld located at the target. The ghost handheld was rendered in horizontal default orientation to prevent subjects from trying to match the orientation (matching the orientation might have changed the task).

The *Screen State* group emulated the typical condition that is commonly used in virtual reality enhanced rehabilitation (Fig 2, left). The *Screen Minimal* group was added for comparison to see if the typically provided depth cues of *Screen State* are worth the development cost, effort, and limitations in artistic freedom for motivational game design (Fig 2, middle). The *Screen Full* group was measured to investigate the open potential between current state-of-the-art (*Screen State*) and what is still feasible using the same hardware (Fig 2, right). In contrast to the above groups *HMD Minimal* and *HMD Full* were the groups recorded to illustrate the open potential with inclusion of head-mounted displays. The rendering details were the same as for the respective screen groups (Fig 2, middle and right). *HMD Full* should show the limitations of what is currently feasible. *HMD Minimal* was added to investigate whether the development cost, effort, and limitations in artistic freedom for additional depth cues is still justified when using a head-mounted display.

### Subjects

In total, 56 healthy volunteers were recruited and pseudo-randomly assigned to the different experimental groups. Recruitment, randomization, and approval by the ethics commission was performed in two phases.

In the first phase, 17 healthy volunteers were recruited and pseudo-randomly assigned to either *Screen State* or to *HMD Full*. Assignment was performed based on a randomly permuted list, ensuring approximately equal group sizes. The experimental protocol and subject recruitment was approved by the ETH Zurich Ethics Commission (EK 2014-N-21).

In the second phase, 39 healthy volunteers were recruited and pseudo-randomly assigned. Assignment was performed based on a randomly permuted list, ensuring the study to have 10 subjects per group at the end. To be allowed to continue with the same experimental protocol, and to recruit additional subjects, a renewed Ethics approval was obtained by the ETH Zurich Ethics Commission (EK-2017-N-13).

Subjects did not have any experience performing our reaching task prior to the experiment. However, some subjects correctly guessed the experimental hypothesis during the trial. All subjects were required to have a range of motion compatible with the experiment, to be 18 to 40 years old, to participate in sports for at least half an hour per week, and to report no non-corrected sight or hearing defects. All subjects had to perform the task with their right hand. Subjects’ dominant hand was determined with the Edinburgh Handedness Inventory [15]. Left-handed and ambidextrous subjects were excluded from analysis. A limit of ±30 in the Edinburgh handedness score was applied to identify ambidextrous subjects as suggested in [16].

In the first phase, one left-handed and two ambidextrous subjects were excluded from data analysis. Their exclusion was corrected for in the randomly permuted assignment list for the second phase. In the second phase, one left-handed and one ambidextrous subject were excluded. Additionally, a third subject, who reported a lack of stereo-vision due to irreversible eye damage was excluded in the second phase. The exclusion in the second phase was corrected by recruiting more subjects with coin-tossed group assignment. In total, data from 50 subjects (10 per group, 12 females in total, mean age 26.6 years, SD 5.0 years) meeting the inclusion criteria were acquired for evaluation.

The depicted individual in both Fig 1 and in the S1 Video is the corresponding first author. The individual in this manuscript has given written informed consent (as outlined in PLOS consent form) to publish these case details.

### Statistical evaluation

Statistical evaluation was performed in Matlab (R2017a) *[The MathWorks Incorporated, Natick, Massachusetts, USA]*. The significance level for all tests was fixed to *α* = 0.05. Statistical tests with a *p* < 0.1 were understood and reported as trends.

For each subject, one average time to target over reference distance was calculated based on all of his/her valid (non early start) movements. For the resulting 50 subject averages (5 groups, 10 subjects each), a Levene test for the assumption of equal variance was performed. In case variance between the groups was significantly different, a non-parametric Kruskal-Wallis test was performed instead of a one-way ANOVA. Multiple comparisons were corrected with a Tukey-Kramer post-hoc test.

For the secondary outcomes, the same procedure was applied to investigate group differences: Creating subject averages, testing the assumption of equal variance between groups, applying one-way ANOVA or Kruskall-Wallis respectively, and finally correcting for multiple comparisons using Tukey-Kramer.

To obtain a more detailed understanding on the primary outcome’s dependencies, a linear mixed model analysis was performed for the primary outcome variable time to target on the level of each individual movement. Candidate linear mixed models were fit by maximum likelihood. The linear mixed model analysis was started with a global intercept and a subject-specific random effect (1|*subject*) correcting for within-subject correlation. The model finding process was continued iteratively by adding of fixed effects, and interactions of hypothesized predicting variables. Each extended model had to explain the observed data significantly better than its parent model or was rejected. Likelihood ratio tests (*χ*^2^) were used to test extended models against their parent. For models of equal statistical complexity, the model with higher likelihood was selected. The following predicting variables were considered as predictors: group, target number, and either reference distance or the improved formulation of Fitts’ Index of Difficulty [17]. Fitts’ Index of difficulty was calculated by *fittsID* = *log*_2_(1 + *d*_*ref*_/*w*_*target*_), where *d*_*ref*_ is the reference distance of the target, and *w*_*target*_ the target width. We used only either reference distance or Fitts’ Index of difficulty as predictors, because the target width was constant in our experiment (*w*_*target*_ = 0.04 m table tennis ball). Target number was included to investigate how improvements over time affect the outcome of our study.

For the resulting linear mixed model, the assumption of normally distributed residuals was visually inspected using a normal Q-Q plot. In case of a heavy tailed residual distribution, the analysis was repeated for the log-transform of the primary outcome. ANOVA marginal testing on the residuals was used to report *p*-values for fixed effects and interactions of the resulting model.

## Results

### Evaluated samples and missing data points

Early starts occurred in 23 out of 1450 evaluated movements (29 movements per subject, 10 subjects per group, 5 groups). The majority of subjects (35/50) did not perform any early start, ten subjects performed 1 early start, four subjects performed 2 early starts, and one subject performed 5 early starts. The group with this last subject *HMD Minimal* had the most early starts: 11 out of total 290 movements. In contrast the group *Screen Full* had the least early starts: 1 out of 290 movements. Due to the low numbers of early starts, they were ignored in further evaluation.

The separate raw data logging failed during the experiment for 6 subjects. While the primary outcome time to target was not affected, the secondary kinematic outcomes could only be evaluated for the remaining 44 subjects.

Movements that were shorter than 0.235 s had to be excluded from the automated speed peak detection due to limitations in the applied smoothing, and were removed from evaluation. Additionally, 24 movements were inspected visually and corrected from 0 automatically detected speed peaks to 1 speed peak, due to the deceleration being inside the target sphere and cut off by the segmentation. In total, 1239 movements from 44 subjects were evaluated for number of speed peaks. Hand path ratio was evaluated for 1253 movements in 44 subjects.

### Subject average group differences

**Fig 3:**
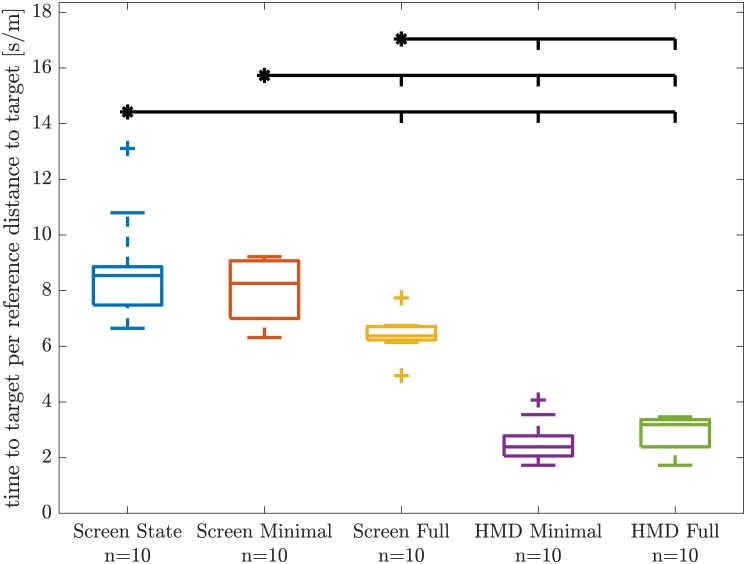
Subject average time to target per reference distance group comparison. The boxes in group color denote median and the 50 coverage intervals. The whiskers indicated ±2.7 standard deviations or 99.3% coverage intervals. Small plus symbols denote the outliers. Horizontal black bars above the boxes denote significant group differences in the Tukey-Kramer post-hoc test; A starred condition was significantly different from the conditions indicated with small vertical bars.

The subjects’ average time to target per reference distance did not show significantly different variances in the Levene test, but indicated a trend towards different variances (*F*_4,45_ = 2.413, *p* = 0.063). One-way ANOVA revealed significant group differences (*F*_4,45_ = 67.980, *p* = 1.712 × 10^−18^). The group *Screen State* was not significantly different to the group *Screen Minimal*, and the group *HMD Minimal* was not significantly different to the group *HMD Full*, all other between group comparisons showed significant group differences in the Tukey-Kramer post-hoc test (Fig 3, Table 2).

**Table 2:** Tukey-Kramer post-hoc group differences in subjects’ average outcomes.

Group 1	Group 2	Time per distance Post-hoc *p*	Number of speed peaks Post-hoc *p*	Hand path ratio Post-hoc *p*
*Screen State*	*Screen Minimal*	0.585	0.999	1.000
*Screen State*	*Screen Full*	1.687 × 10^−9^	0.307	0.489
*Screen State*	*HMD Minimal*	9.922 × 10^−9^	1.412 × 10^−4^	2.093 × 10^−4^
*Screen State*	*HMD Full*	9.922 × 10^−9^	2.104 × 10^−4^	1.348 × 10^−3^
*Screen Minimal*	*Screen Full*	0.014	0.412	0.353
*Screen Minimal*	*HMD Minimal*	9.922 × 10^−9^	2.291 × 10^−4^	5.250 × 10^−5^
*Screen Minimal*	*HMD Full*	9.923 × 10^−9^	3.422 × 10^−4^	4.116 × 10^−4^
*Screen Full*	*HMD Minimal*	1.710 × 10^−8^	0.092	0.052
*Screen Full*	*HMD Full*	7.855 × 10^−8^	0.115	0.159
*HMD Minimal*	*HMD Full*	0.963	1.000	0.991

**Fig 4:**
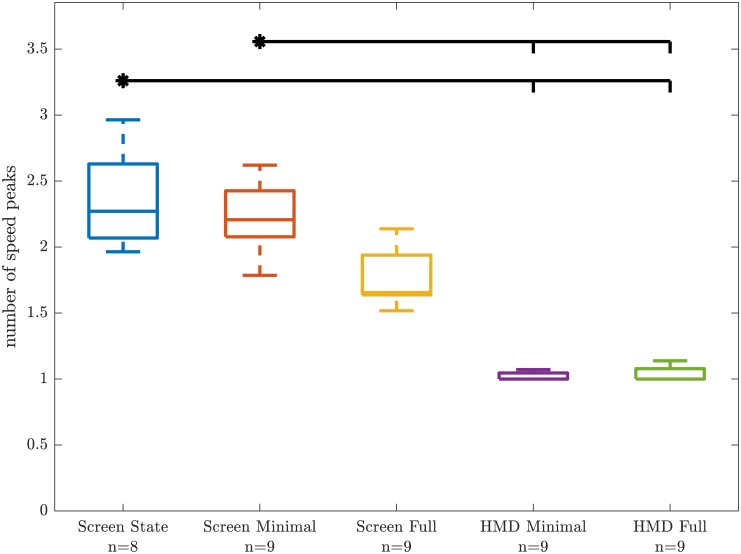
Subject average number of speed peaks group comparison. The boxes in group color denote median and the 50 coverage intervals. The whiskers indicated ±2.7 standard deviations or 99.3% coverage intervals. Small plus symbols denote the outliers. Horizontal black bars above the boxes denote significant group differences in the Tukey-Kramer post-hoc test; A starred condition was significantly different from the conditions indicated with small vertical bars.

The subject average number of peak speeds per movement showed significantly different variance in the Levene test (*F*_4,39_ = 7.990, *p* = 8.342 × 10^−5^). Kruskal-Wallis revealed significant group differences ($\chi_{4,39}^{2}=36.114$, *p* = 2.741 × 10^−7^). In the Tukey-Kramer post-hoc, only the two groups wearing the HMD were significantly different from *Screen State* and *Screen Minimal* (Fig 4, Table 2).

The subject average hand path ratio behaved using a similar pattern as the average number of peak speeds: Levene test failed (*F*_4,39_ = 5.331, *p* = 1.597 × 10^−3^) and Kruskal-Wallis revealed significant group differences ($\chi_{4,39}^{2}=35.461$, *p* = 3.734 × 10^−7^). Also, the same significant group differences in post-hoc as in the average number of peak speeds were found (Table 2).

### Linear mixed model analysis of time to target

Linear mixed model analysis of time to target resulted in a heavy tailed norm Q-Q plot, which violated the assumption for normally distributed residuals. Therefore, the linear mixed model analysis was completed using the *log*_2_-transform of time to target as the dependent variable. Models using the improved formulation of Fitts’ Index of Difficulty as a fixed effect achieved higher likelihood with equal statistical complexity than models directly using reference distance.

**Fig 5:**
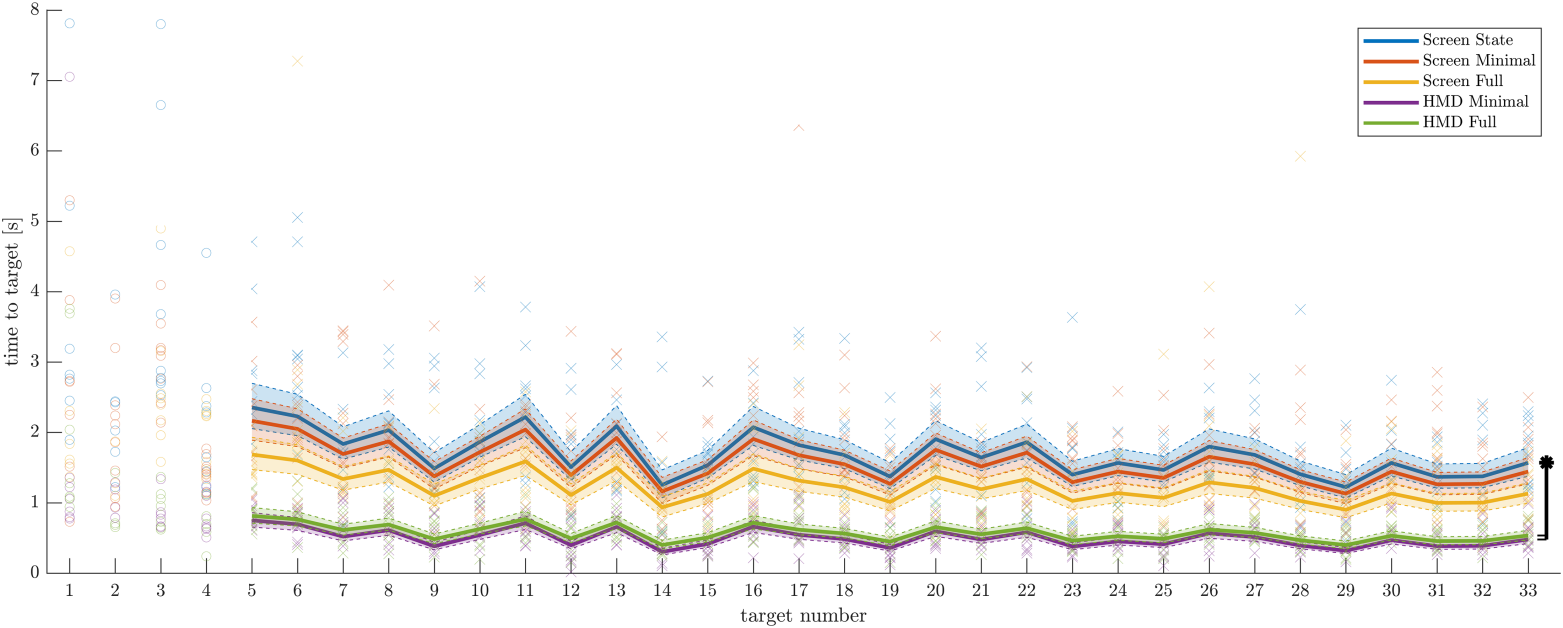
Time to target over target numbers including the final linear mixed models. Raw data of unevaluated targets (number 1 to 4) are illustrated with small circles in respective group color. Raw data of evaluated targets with small crosses. The y-Axis is scaled to fit all evaluated raw data (crosses), four data points of not evaluated data (circles) between 8.5 s and 12 s were cropped. The continuous lines in respective group color denote the subject-unspecific linear mixed model estimate for the current target with dashed confidence bounds. The piece-wise linearity in the plot arises from the dependence on Fitts’ Index of Difficulty, which was arising from different target reference distances. The black vertical bar indicates the significant group offsets between reference level *Screen State* (*) to both HMD groups (indicated with small horizontal black lines).

ANOVA marginal testing on the resulting linear mixed model for the *log*_2_-transform of time to target revealed a significant group main effect (*F*_4,1416_ = 33.048, *p* = 2.215 × 10^−26^), a significant linear dependence on increasing target number (*F*_1,1416_ = 107.37, *p* = 2.665 × 10^−24^), a significant linear dependence on Fitts’ Index of Difficulty (*F*_1,1416_ = 46.255, *p* = 1.526 × 10^−11^), and a significant interaction between group and Fitts’ Index of Difficulty (*F*_3,1416_ = 2.701, *p* = 0.029). The raw data points of achieved time to target over the target numbers, as well as the final models with are illustrated in Fig 5. The model based on 1427 observed movements. Model coefficients for *Screen State* as the base level group are provided in Table 3.

**Table 3:** Linear mixed model coefficients.

**Fixed effect name**	**Estimate**	**SE**	**tStat**	***p*-value**	**Lower**	**Upper**
*globalIntercept*	−0.036	0.131	−0.276	0.783	−0.293	0.220
*group*_*ScreenMinimal*_	−0.070	0.182	−0.384	0.701	−0.427	0.287
*group*_*ScreenFull*_	−0.260	0.181	−1.435	0.151	−0.616	0.095
*group*_*HMDMinimal*_	−1.643	0.183	−8.994	7.445 × 10^−19^	−2.001	−1.285
*group*_*HMDFull*_	−1.205	0.182	−6.631	4.718 × 10^−11^	−1.561	−0.848
*targetNumber*	−0.012	1.114 × 10^−3^	−10.362	2.665 × 10^−24^	−0.014	−9.356 × 10^−3^
*fittsID*	0.411	0.060	6.801	1.526 × 10^−11^	0.292	0.529
*group*_*ScreenMinimal*_: *fittsID*	−6.103 × 10^−3^	0.085	−0.071	0.943	−0.174	0.161
*group*_*ScreenFull*_: *fittsID*	−0.032	0.085	−0.374	0.709	−0.199	0.135
*group*_*HMDMinimal*_: *fittsID*	0.216	0.086	2.513	0.012	0.047	0.385
*group*_*HMDFull*_: *fittsID*	0.061	0.085	0.719	0.472	−0.106	0.229
**Random Effect Name**	**Estimate**				**Lower**	**Upper**
(1|*partNumber*) STD	0.185				0.149	0.231
Residual STD	0.349				0.336	0.363

## Discussion

### Group differences in reaching performance

#### Missing group differences between *Screen State* and *Screen Minimal*

The missing group differences between *Screen State* (Fig 2, left) and *Screen Minimal* (Fig 2, middle) indicate that the depth cues typically realized with two-dimensional screens do not provide any measurable benefit in reaching performance (Fig 3). The only depth cue available for subjects in *Screen Minimal* was the relatively referenced object size of the target sphere. Either this relatively referenced cue of retinal image size was the dominant cue for 3D reaching movements, such that the other provided depth cues would not matter, or all other provided depth cues from *Screen State* remained too abstract for our subjects to use intuitively. For the latter case, subjects in *Screen State* could possibly learn to use these depth cues if they were trained for a longer time under this condition and ultimately outperform subjects in the *Screen Minimal* group. However, no group differences in improvements over target numbers (potential learning) was observed in our linear mixed model analysis (Fig 5). Therefore, it is inconclusive how many trials are be required for learning to occur and become relevant.

We conclude that for 2D screen setups, state of the art renderings were not beneficial for depth perception when compared to abstract renderings.

#### Group *Screen Full* outperforms other *Screen* groups

The additional provision of an absolutely referenced object of known size resulted in a significant lower time to target per reference distance (Fig 3).

If an increased depth perception due to this absolutely referenced object of known size was responsible for the performance improvements remains questionable. The display of the tracked handheld within the virtual environment could have raised the perceived spatial immersion and general presence in the virtual environment. An increase in immersion could cause the other available depth cues to become intuitively usable, or could increase subject motivation [18] confounding the observed reaching performances.

Placing ghost handles at target positions is a severe design limitation for 3D virtual environments: If multiple valid targets were displayed at the same time, placing ghost handhelds may become infeasible. Potentially, the benefits of absolutely referenced objects of known size could also be achieved without ghost handhelds, e.g. a yellow ping-pong ball of target-sphere size mounted above the screen, or given to the subject to hold in his/her off-hand could have the same effect.

We conclude that for 2D screen setups, providing both handheld rendering, and handheld ghost objects at the targets, reduced difficulty for users when interacting with the virtual environment. We strongly suggest to include absolutely referenced objects of known size into virtual environment design for 2D screens.

#### *HMD* groups outperform *Screen* groups

While we expected the head-mounted display (HMD) groups to perform better, we were surprised by how strong the observed effect was (Fig 3). Both stereopsis and motion parallax by tracked head movements could have been responsible for improved depth perception.

Besides improved depth perception, the increased field of view and the generally raised immersion of the HMD may have improved the observed reaching performances. One interpretation of our results could be that using the HMD was so immersive for subjects that they could rely solely on subconscious motor control, meaning they only had to think actively about where to move the handheld but not about the movement execution itself. In contrast, subjects using the 2D screens effectively had to consciously use visuomotor control to synchronize both their hand movements in real world and displayed movements on the screen. Thus, this additional mental load of actively using visuomotor control and mapping from real world to virtual world could have been partially responsible for the lower performances.

Besides lower demands in visuomotor control, motivation bias due to increased immersion might also have contributed to the higher observed performances. We tried to create a sham-controlled group: Subjects wearing the HMD, seeing a rendered virtual screen, which displayed the scenery in 2D. Due to the limited horizontal resolution of the HMD, the resulting 2D resolution of the virtual screen was too poor (< 260 pixels horizontally) to be reasonably tested with an experimental group.

The improved performance of *HMD* groups was not only based on greater movement speeds, but also fewer speed peaks and shorter movements (see Table 2, Fig 2). While lower motivation in *Screen* groups could explain slower movements, it seems hard to explain how lower motivation would lead to longer and less efficient movements. Therefore, we were convinced that motivation bias could be a contributing factor, but not solely responsible for the observed differences between groups.

We conclude that using head-mounted displays simplifies 3D reaching independent of the rendered level of detail. Using a HMD with abstract renderings (Fig 2, middle) resulted in better performance than detailed depth cue renderings (Fig 2, right) displayed on a 2D screen.

#### Missing group differences between *HMD Minimal* and *HMD Full*

We have not observed any significant differences between the group *HMD Minimal* and *HMD Full* (Fig 3). Compared to all other groups, both *HMD* groups achieved extremely good performance. Potentially the difficulty of our 3D reaching task showed a floor effect: If both conditions with the HMD provided sufficient reliable depth information and were realistic enough, the difficulty of performing 3D reaching movements in the virtual would reach a lower limit, i.e. was as simple as performing real reaching movements. If both conditions reached that lower limit in difficulty, the additionally rendered depth cues would not be necessary.

Subjects in the *HMD Minimal* group seemed to perform slightly better than subjects in the *HMD Full* group (Fig 3). This might be an indication that the additional rendered depth cues were not only unnecessary, but could also be distracting. However, this effect is within the noise level and far from statistical significance given the investigated group sizes.

We conclude that stereopsis, motion parallax, and relatively referenced size of the yellow target sphere provided sufficient depth information when using a HMD, such that additional virtually recreated visual depth cues had no further positive effect.

### Limited depth cues affect number of speed peaks and hand path ratio

Both number of speed peaks and hand path ratio both showed the same trends (Fig 4): Subjects using the HMD achieved results towards one single speed peak and a hand path ratio of around one, which for both metrics would reflect the expected theoretical optimum. Subjects of the *Screen State* and *Screen Minimal* group showed significantly more speed peaks and higher hand path ratios. Subjects of the *Screen Full* group were somewhere in between, but differences to both the HMD groups and the the other screen groups were not significant (Table 2).

The achieved optimal minima of both *HMD* groups in these quality of movement metrics may be an indication that HMD technology is good and immersive enough to enable realistic movements based on natural, subconscious motor control. HMD setups seem to enable a more direct measurement of abilities or impairments in motor control than conventional 2D screen setups. However, our study was performed with healthy, relatively young individuals. If our findings can translate to elderly subjects or patients with sensory deficits needs to be verified for the individual target population.

*Screen State* and *Screen Minimal* did not achieve optimal minima in these quality of movement metrics. These display setups have limitations that cause artifacts in the executed movements. Therefore, quality of movement metrics obtained with instrumented setups that use similar displays should be interpreted carefully. Optimal performances that can be expected with the given setup probably do not align with expected performances from theoretical limits. In our study, theoretically expected performances could not be achieved, even by healthy young adults. Our results underlined the importance of setup-specific normative data for the interpretation of assessments targeting function or disability.

Since 2D screen setups already limited how naturally reaching movements could be performed, the transfer from training natural movements with such setups to reality might be limited as well. For instrumented rehabilitation devices that rely on visual information of 2D screen setups (e.g. in [9, 19]), we would suggest to focus on motivating, highly repetitive, and intensive training, but to a lesser extent on trying to directly mimic activities of daily living. Also, rehabilitation robots (e.g. ARMin [20]) can enforce natural movements with haptic interaction, e.g. by constraining the patient’s movement or by supporting forces. Whether haptic feedback from such support or enforcement could partially compensate limitations of the 2D screens remains unanswered. However, if mimicking activities of daily living with robotic support might still be superior to abstract movement training for severely affected patients would need to be confirmed experimentally. We cannot provide a suggestion on this topic based on our study.

The *Screen Full* group was not significantly different to any other group. This could be a sensitivity issue that may be possibly resolved with a greater number of subjects. We would expect these outcomes to be more sensitive if subjects were instructed to and rewarded for moving as smoothly and in a movement as straight as possible, instead of the given instruction to move as quickly as possible. However, for such an alternative experimental design, controlling for an equal speed accuracy trade-off between the subjects becomes more challenging and the interpretation of the quality of movement metrics could become less straight forward.

### Linear mixed model analysis supports experimental design

Even though an expected improvement resulted over the target numbers, it did not confound our results and primary evaluation. Improvement over target numbers seemed relatively small and no significant interaction between test group and this improvement resulted (Fig 5, Table 3).

We could show that our time to target is proportional to Fitts’ index of difficulty, and that even though we applied only a single constant target width, Fitts’ index of difficulty was a better predictor than distance. To obtain reasonable residuals for our linear mixed model fit we needed to log-transform our outcome time to target. The classical Fitts’ Law equation that we would have expected does not have this log-transform of time. However, we did not consider such a difference to the classical Fitts’ Law to be a problem, because our task involved an additional 2 seconds dwell time inside the three dimensional target, and our target size was constant.

The significant group effect and interaction between group and Fitts’ index of difficulty seem to confirm our primary evaluation of time to target per reference distance. The non-significant group main effects in Table 3 should not be misinterpreted as missing group differences. Firstly, groups were only compared to tested reference level *Screen State*, e.g. differences between *Screen Full* and *HMD Full* cannot be read out. Secondly, due to the significant interaction between group Fitts’ index of difficulty, we have two independent parameters between the groups, which makes interpretation nontrivial.

### Limitations and outlook

Our primary goal was to investigate the potential of better depth cue rendering or new HMDs to improve 3D instrumented rehabilitation. Since our experiment was performed with young healthy adults, transfer of our results to an elderly target population or subjects with sensory deficits may be limited. We do not know if elderly that are less accustomed to computer screens would show the same behavior when exposed to the different rendering scenarios and how they would react to head-mounted displays.

Using HMDs for therapy still faces challenges: Current HMDs are consumer products that were not intended or certified for medical use, and cleaning and disinfection possibilities are limited. Weight of current high performance HMDs could cause severe acceptance problems for wearing them over prolonged time, especially for elderly or impaired subjects. The complete isolation from real world view could lead to acceptance problems from both patients and therapists. Also, isolation from real world view combined with the relatively bulky size of both rehabilitation robots and current HMDs could increase the risk of patients hitting themselves during therapy.

However, with a further down-scaling of hardware size and weight, the development of see-through head-mounted displays and augmented reality systems, such limitations could be overcome. Also, screen based solutions incorporating head tracking and stereopsis could provide similar immersion and depth perception to HMDs without their current drawbacks. However, we believe already current HMD technology could enhance rehabilitation for medium to mildly affected patients to a similar extent as rehabilitation robots for severely affected patients.

The use of the HTC Vive as a tool for research has been questioned in a recent work evaluating tracking accuracy and precision for room-scale setups for a 8 m×4 m work-space [21]. In our experiment, the HTC Vive was set up in a seated-only condition, covering a work-space of approximately 1.2 m×0.8 m that was fully covered by both Lighthouse base stations. For such a condition, we are not aware of a similar extensive evaluation of accuracy and precision. Since we have used the same measurement setup in all groups, we are convinced that measurement inaccuracies were present and comparable in all subjects and did not bias our between group comparisons.

## Conclusion

Head-mounted displays provided immersion and depth perception on a level that proved sufficiently realistic for healthy young adults to perform natural reaching movements. In contrast, two-dimensional (2D) screen setups lowered performance and altered hand trajectories. Virtually recreated visual depth cues had a minor impact. We conclude for both head-mounted display and 2D screen setups, motivational aspects should be favored over depth cues where compromises are necessary in designing virtual environments for training.

Performance or impairment assessments for 3D reaching function obtained with 2D screen setups should be interpreted with care. Movement artifacts due to limited depth perception or immersion prevent even healthy young adults from reaching expected theoretical limits. We are convinced that head-mounted displays hold an immense potential to improve upper limb movement training and assessments. However, therapeutic use of head-mounted displays seemed still limited due to size, weight, and isolation from real world view.

## Supporting information

S1 Fig Subject average hand path ratio group comparison. The boxes in group color denote median and the 50 coverage intervals. The whiskers indicated ±2.7 standard deviations or 99.3% coverage intervals. Small plus symbols denote the outliers. Horizontal black bars above the boxes denote significant group differences in the Tukey-Kramer post-hoc test; A starred condition was significantly different from the conditions indicated with small vertical bars.
(EPS)

S1 Video Demonstration video of the task protocol. The video shows the first author completing the task with all 33 targets in the *HMD Full* condition. The measurement setup and the screen rendering is visible in the back. The video is post-processed with variable and increased replay speeds (x1 to x32) to reduce playback time to 1 min and 17 s. The replay factor is labeled in the top left corner and the target number in top middle. A false start is performed at 00:54 s for target number 18.
(MP4)

S1 Table More detailed information on depth cue implementation and rationale. The supplementary S1 Table was added to give some more detailed information and rationale on the different depth cues from Table 1.
(PDF)

S1 Protocol Investigator’s study protocol. The supplementary S1 Protocol is the checklist according to which the investigator instructed the subject. Dependent on the subject’s preference, the instruction was given in English or German.
(PDF)

S1 Dataset Result data container. The supplementary S1 Dataset container (.zip file) contains two Matlab^®^ tables (saved as .mat files). “averageResultsTableFile.mat” contains the outcome data of subject means required for the between group analyses (Fig 3, Fig 4, Table 2, and S1 Fig). “resultsTableFile.mat” contains the outcome data for each individual movement required for linear mixed model analysis (Fig 5 and Table 3).
(ZIP)
