# Supplementary material for: Missing depth cues in virtual reality limit performance and quality of three dimensional reaching movements
Source: PLoS One. 2018 Jan 2;13(1):e0189275. doi: 10.1371/journal.pone.0189275 (PMC5749675; doi:10.1371/journal.pone.0189275)
Supplement: S1 Table — The supplementary S1 Table was added to give some more detailed information and rationale on the different depth cues from Table 1. (PDF) [file pone.0189275.s003.pdf]

S1 Table. Additional Information on Depth Cue Implementation and Rationale

Depth cue	Implementation	Details	Comments and Rationale
**Aerial perspective**	Virtual fog.	Aerial distance was realized in the standard asset "Global Fog" provided in our development environment Unity 3D. It is a camera-based exponential fog with a chosen exponent of 2.5 and maximum fog saturation on a value of 0.25. Fog color was to grey (RGBA 0.5, 0.5, 0.5, 1).	Even though we do not expect aerial perspective do be a significant depth cue for locating close objects in reachable space itself, we have chosen to include it for the *Screen State* condition following reasons:<br>• We consider the implementation of aerial distance as standard, since it can be realized without relevant effort by prefabricated assets in most game development environments.<br>• For an open scenery with visible horizon as used in our experiment, the lighting conditions appear very artificial/abstract without any fog or aerial distance.<br>• To our research question the inclusion of a "probably negligible" cue in the state-of-the-art condition is tangential. A better state-of-the-art would strengthen our results for both: Showing no difference between state-of-the-art compared to the minimal condition and showing that state-of-the-art can be improved. In contrary, exclusion of such a "probably negligible" cue to the state-of-the-art group would need to be reasoned and could be criticized to eventually (if not negligible) contribute to our result.
**Linear perspective**	White parallel lines on the scenery ground.	The white parallel lines on the scenery ground were implemented by using a ground texture (wrap mode repeat) with white vertical borders on the left and right side.	Linear perspective with the lines on the ground do not directly give any cues on the distance to the target sphere or handle, but provide a static depth reference for objects located on the ground or for shadows.

**Motion parallax** (scenery motion)	Constant virtual wind moving blades of grass, closer blades of grass appear to be moving more.	Blades of grass and their movement were realized with the Unity 3D tree editor and Unity 3D global scenery wind. Detail settings were hand tuned to obtain a blade tip movement that seemed pleasant, non-disturbing, and in a reasonable amplitude.	The resulting movement amplitude on the display of blades that are similar in size, share the same underlying physics and wind provide a depth relation between the grass tips. We were convinced that this depth relation between the grass tips also helps on mapping depth of the scene into the vertical direction better than just the static depth reference due to their position on the ground. Also, tip movement in conjunction with the shadows of the tips when moving back and forth on the ground was assumed to help to improve localization of other shadows in the scene over just static positions.<br>Most rehabilitation games or assessment environments do probably not contain such a motion parallax due to scenery motion. However, we have decided to include this cue since it can relatively easily be included with modern game development environments and can be realized interdependently of desired realism of the scene. Additionally, the same reasoning as for aerial distance applies: testing a better state-of-the-art condition could strengthen our results, while for a worse condition justifications would be needed.
**Occlusion**	Blades of grass, that may partially occlude each other, target, or pointer.		Occlusion can give more than just qualitative information on the depth relation between objects dependent on the visible v.s. covered surface of the object in the back, if the relative sizes of the objects are known.
**Shadows**	Shadows of grasses, pointer, target, handhelds, and ghost handheld.	Shadows were realized by using the default Unity 3D Sky-box for scene lighting, which appears to be a combination of ambient and a distant light source. Shadows can be turned off completely for any light source in Unity 3D.	Shadows provide not only depth relation by indicating the position on ground reference, but also relative depth between shadows, and the size of the shadows can also give a cue if a relative sizes of the objects are known.
**Texture gradients**	Grey texture at scenery ground, where a more detailed rendering is visible closer to the subject.		Similar to the linear perspective cue, the grey honeycomb-like ”kaleidofloor” texture applied to the ground was assumed to only provide a static depth reference for objects located on the ground or for shadows.

**Retinal image size** (absolute)	Objects of absolutely known size at both, pointer position by showing virtual black handheld, and at target position by white ghost handheld.	The 3D model of the HTC Vive handheld reflecting "real" size was provided by the OpenVR library.	Subjects know the exact size of the handheld since they see (or have seen before putting on the HMD) it and hold it in their hands (proprioception on their fingers). If this object of "truly" known size is now moved in the virtual world and changes size dependent on the depth - the amount of size change of the known object per hand movement provides a learn-able absolute depth reference for the virtual scene. However, the addition of the ghost object a the target was assumed to be the major deal here. Having two identically sized objects at different depths appearing in different sizes was assumed to provide an absolute, very quantitative relation between their position, simplifying the formation on any visuomotor mapping between perception of VR and hand movements that might be required to to fulfill the task substantially.
**Retinal image size** (relative)	Unrelated objects of relatively known size, constant sized pointer and constant sized target sphere.		While the subjects did not know initial size of pointer and target, they had a 1 minute familiarization time to move the pointer around the work-space and learn its relative size change to distance. Additionally, they had the four trial targets to learn the size of the sphere in relation to the pointer. However, no absolute clues were provided how large sphere or pointer was. Since these objects were not the same, retrieving quantitative information due to their appearing sizes on the screen is more challenging and less direct than in the case with two equal, absolutely known objects.<br>While sometimes "handheld avatars" of absolutely sizes are used in rehabilitation games or assessments (e.g. robot end pieces), relative or generic "hand avatars" seem to be the gold standard, or more common. We have not yet seen any example that used absolute handheld avatars and absolute ghost objects at desired targets.
**Motion parallax** (subject motion)	Virtual scene is rendered from the current viewing position and angle, which can be tracked by the HMD, close objects seem to move more.	Scene rendering from the viewer angle was realized by using the prefabricated camera objects in Unity 3D provided by the OpenVR library for the HTC Vive headset.	
**Stereopsis**	Individual screens for each eye in the HMD providing different images for both eyes.	Stereopsis was realized by using the prefabricated camera objects in Unity 3D provided by the OpenVR library for the HTC Vive headset.	
