# Supplementary material for: Missing depth cues in virtual reality limit performance and quality of three dimensional reaching movements
Source: PLoS One. 2018 Jan 2;13(1):e0189275. doi: 10.1371/journal.pone.0189275 (PMC5749675; doi:10.1371/journal.pone.0189275)
Supplement: S1 Protocol — The supplementary S1 Protocol is the checklist according to which the investigator instructed the subject. Dependent on the subject’s preference, the instruction was given in English or German. (PDF) [file pone.0189275.s004.pdf]

✓	
	1. Ensure curtains are installed on windows.
	2. Start virtual environment, calibrate workspace, and set group parameters.
	3. Set volume- PC: 80, speakers: 3.5 marks.
	4. Ensure seat and monitors are at marked positions.
	5. Welcome participant. Explain purpose of study, risks (Cybersickness and hitting self or table), that there is no compensation, and that participant is free to withdraw at any time. Ask participant if they have any questions; answer if necessary.
	6. Have participant complete consent form and pre-trial questionnaire.
	7. **HMD only:** Measure IPD and adjust.
	8. **HMD only:** Help participant to put on HMD and re-adjust for comfort and security if necessary.
	9. Read:<br><br>"Here you see a virtual environment. I will now hand you the controller."<br>"Da siehst du eine virtuelle Umgebung. Ich werde dir jetzt den Controller geben."
	10. Hand controller to participant.
	11. Read:<br><br>"Notice that as you move the controller in real space, it moves similarly in virtual space. Please take a minute to explore your full range of motion."<br>"Beachte, dass wenn du den Kontroller in der Realität bewegst, er sich ebenso in der virtuellen Umgebung bewegt. Nimm dir Zeit um deinen vollen Bewegungsumfang zu erforschen."
	12. Allow one minute of familiarization time.
	13. Read:<br><br>"Afterwards you will see a yellow sphere. When you touch the pink pointer to the yellow sphere, the pointer will turn green."<br>"Nachher wirst du einen gelben Ball sehen. Wenn du mit dem pinken Zeiger den Ball berührst, wird der Zeiger grün."<br><br>"After ten seconds, the sphere will move to a new position. Take note of this position, but do not move until the 'Wait' message disappears."<br>"Nach 10 Sekunden, wird sich der Ball an eine neue Position bewegen. Nimm die neue Position zur Kenntiss, aber bewege dich nicht bis das 'Warte' Zeichen verschwindet."<br><br>"Wait for wait message to disappear. You will hear a "peep peep peep piip" sound and feel a short vibration, then you can move."<br>"Warte bis das warte Zeichen verschwindet. Du wirst ein "peep peep peep piip" Geräsch hören und eine kurze Vibration spüren."

continues on reverse

	"Then move as quickly as possible to the new position. Hold the controller there for two seconds."<br>"Dann bewege dich so schnell es geht an die neue Position. Und halte den Zeiger für 2 Sekunden dort."<br><br>Wait for success.<br>Warte auf die Bestätigung.<br><br>"Then the sphere moves back to the rest position. Return slowly to the rest position, minding not to hit the table."<br>"Dann wird sich der Ball zurück auf die Ruhe Position bewegen. Gehe langsam zürück zur Ruhe Position und pass auf, dass du nicht auf den Tisch haust.<br><br>"This process will repeat for 33 targets total. And you can see your progress in the top of the screen. Do you have any questions?"<br>"Dieser Prozess wird sich 33 mal wiederholen. Und du wirst deinen Vortschritt oben am Bildschirm sehen. Hast du irgendwelche Fragen?"<br><br>"Then we start."<br>"Dann starten wir jetzt."
	14. Activate target.
	15. Observe reaching for first four targets (maximum radius). If unsuccessful, exclude participant.
	16. Continue through remaining targets.
	17. Collect controller and (if applicable) HMD from participant.
	18. Stop virtual environment and save 2 log files to local rawData folder.
	19. Copy local rawData folder to network location.
	20. Ask participant to complete follow-up questionnaire.
	21. Thank participant.

#: ____________________

Participant: ____________________

Date: ____________________

Group: ❑ 1 HMD Full ❑ 2 Screen State ❑ 3 Screen minimal
❑ 4 Screen Full ❑ 5 HMD minimal

Time started: ____________________

Time finished: ____________________

Hand: ❑ L ❑ R
